# Clinical Characteristics and In-Hospital Outcomes in Patients with Iliopsoas Abscess: A Multicenter Study

**DOI:** 10.3390/jcm12082760

**Published:** 2023-04-07

**Authors:** Yi-Chih Lee, Jhih-Jin Li, Chien-Han Hsiao, Chieh-Ching Yen

**Affiliations:** 1 Department of Emergency Medicine, Chang Gung Memorial Hospital, Linkou Branch, Taoyuan 33305, Taiwan; 2 Department of Linguistics, Indiana University, Bloomington, IN 47405, USA; 3 Department of Emergency Medicine, New Taipei Municipal Tucheng Hospital, New Taipei City 23652, Taiwan; 4 Department of Emergency Medicine, Jen-Ai Hospital Dali Branch, Taichung 412224, Taiwan

**Keywords:** iliopsoas abscess, in-hospital mortality, length of hospital stay (LOS)

## Abstract

(1) Background: Iliopsoas abscess (IPA) is usually overlooked due to its nonspecific symptoms and signs. The resulting delayed diagnosis and treatment can increase morbidity and mortality. The purpose of the present study was to identify the risk factors for the unfavorable outcomes associated with IPA. (2) Methods: We included patients who presented to the emergency department and were diagnosed with IPA. The primary outcome was in-hospital mortality. Variables were compared, and the associated factors were examined with Cox proportional hazards model. (3) Results: Of the 176 patients enrolled, IPA was of primary origin in 50 patients (28.4%) and of secondary origin in 126 (71.6%). Skeletal origin was the most common source of secondary IPA (*n* = 92, 52.3%). The most common pathogens were Gram-positive cocci. Eighty-eight (50%) patients underwent percutaneous drainage, 32 (18.2%) patients underwent surgical debridement, and 56 (31.8%) patients received antibiotics. Multivariate analyses indicated that age > 65 (year) (HR = 5.12; CI 1.03–25.53; *p* = 0.046), congestive heart failure (HR = 5.13; CI 1.29–20.45; *p* = 0.021), and platelet < 150 (103/μL) (HR = 9.26; CI 2.59–33.09; *p* = 0.001) were significant independent predictors of in-hospital mortality in Model A, while the predictors in Model B included age > 65 (year) (HR = 5.12; CI 1.03–25.53; *p* = 0.046) and septic shock (HR = 61.90; CI 7.37–519.46; *p* < 0.001). (4) Conclusions: IPA is a medical emergency. Our study reported that patients with advanced age, congestive heart failure, thrombocytopenia, or septic shock had a significantly higher risk of mortality, and the recognition of the associated factors may aid in risk stratification and the determination of the optimal treatment plan for IPA patients.

## 1. Introduction

The iliopsoas abscess (IPA), a suppurative collection within the compartment of the psoas and iliacus muscles, was first reported by Mynter et al. in 1881 [1]. IPA is an easily overlooked but potentially serious infectious disease due to its insidious onset and rarity [2,3]. The classic triad of fever, flank pain, and hip movement limitation—referred to as the psoas-muscle sign—is only present in 30% of its patients, resulting in difficult diagnosis [4]. Delays in the appropriate treatments may lead to increased morbidity and mortality [5,6].

Given that the iliopsoas muscles are adjacent to certain organs, they are susceptible to infections from these contiguous structures via the blood supply and overlying lymphatic system [5]. Primary IPA occurs when the causative organism spreads through the bloodstream or lymphatic system from a distant site of infection, and it accounts for approximately 30% of all IPA cases. In contrast, secondary IPA develops as a result of direct extension of an adjacent infectious process into the iliopsoas muscle, and it accounts for the remaining 70% of cases [7]. The associated risk factors included diabetes, intravenous drug use, renal failure, human immunodeficiency virus (HIV) infection, and other forms of immunosuppression. Trauma and hematoma formation can lead to the development of psoas abscesses, especially for secondary IPA [8]. The mortality rates were significantly different among the two categories of IPA, with approximately 2.4% for primary IPA, and up to 18.9% for secondary IPA [9].

In recent years, because imaging modalities, e.g., gallium-67 inflammation scan, computed tomography, and magnetic resonance imaging, have undergone major advances in their speed, resolution, and multiplanar capacity, a growing body of literature reports cases of IPA [2,10,11]. Besides the improved diagnostic techniques, the aging population and the increase in immunocompromised hosts contribute to the rise of the incidence rate of IPA as well [2,12].

The first line treatment of IPA typically involves administering broad-spectrum antibiotics that provide coverage against potential microorganisms, with a focus on Staphylococcus aureus (*S. aureus*) [7]. Surgical drainage, by acclamation, was the treatment of choice, and previous studies have reported quicker recovery following the open procedure [3]. More recently, image-guided percutaneous drainage (PCD) has been established as another effective and safe alternative procedure [3,7].

Even though there are established treatment procedures, the mortality rate of IPA is still high due to the difficulty of early recognition. Although several studies have emphasized the importance of the early recognition and timely treatment of IPA, the prognostic factors for poor outcomes have yet to be documented in detail due to the small number of patients in the published studies. The aim of this study was to evaluate the clinical characteristics and identify the predictors of poor outcomes in patients with iliopsoas abscess.

## 2. Materials and Methods

### 2.1. Study Design and Setting

This study was a retrospective, multicenter, observational study that utilized regularly collected electronic medical records (EMRs) from the emergency departments (EDs) of six hospitals in Taiwan that sharing the same EMR system. These hospitals included two tertiary medical centers and four regional hospitals. The combined capacity of these study sites exceeded 10,000 beds, and the EDs recorded an annual patient visit count of more than 500,000. The data collection period extended over six years, from 1 January 2016 to 31 December 2021. This study was conducted in accordance with the Declaration of Helsinki and received approval from the institutional review board of the Chang Gung Medical Foundation (IRB no. 202201959B0) with qualification for a waiver of informed consent.

### 2.2. Patient Selection and Data Collection

Through searching the electronic medical records (EMRs) during the study period, all the patients diagnosed with iliopsoas abscess in the EDs were first identified. Patients with an age under 18, incomplete medical records, or duplicated data were excluded. The patients selected by the EMRs were further reviewed by three physicians (L.-J.J., L.-Y.C., Y.-C.C.) for their inclusion eligibility.

**Figure 1:**
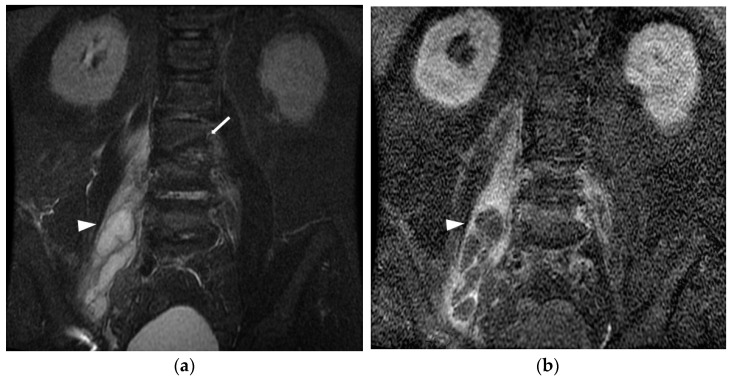
Pyogenic spondylodiscitis complicating iliopsoas abscess in a 58-year-old male. (**a**) Magnetic resonance (MR) image of L-S spine of coronal short T1 inversion recovery (STIR) sequence showed cortical discontinuities and erosions of endplates (arrow) of L2, L3 vertebras with hyperintense of right iliopsoas muscle associated with intramuscular loculated fluid collections (arrowhead). (**b**) Contrast-enhanced MRI T1 weighted image (WI) with fat suppression showed prominent rim enhancement of loculated fluid collection in the right iliopsoas muscle, which was compatible with intramuscular abscess of the right iliopsoas muscle (arrowhead).

A diagnosis of iliopsoas abscess was made if the patient showed the clinical symptoms of iliopsoas abscess and met one of the following criteria. (1) One or more positive culture or gram stain on iliopsoas compartment aspiration or surgical specimens was detected; or (2) radiological findings—including evidence from computed tomography (CT) or magnetic resonance imaging (MRI)—revealed the presence of an iliopsoas abscess, which was consistent with the patient’s clinical presentation. The characteristics of an iliopsoas abscess in CT or MRI typically include: (1) enlargement of the involved muscle; (2) rim enhancement around the abscess with decreased density in the center; and (3) the presence of gas within the abscess, visible as an air–fluid interface or small pockets of air [1]. The images were reviewed by experienced radiologists to ensure accurate diagnosis of iliopsoas abscess (Figure 1).

For each identified patient, the demographic information (i.e., age and sex), initial vital signs upon admission, and comorbidities (i.e., hypertension, diabetes mellitus, chronic kidney disease, malignancy, intravenous drug abuse, coronary artery disease, liver cirrhosis, prior stroke, congestive heart failure, HIV infection, and prior tuberculosis (TB), were retrieved. Information regarding the initial presentations, site of involved iliopsoas muscle, pattern, and size of abscess formation, laboratory findings, the source of origin, organisms identified from blood or abscess cultures, treatment modalities, and length of hospital stay were collected. Laboratory findings, which included the results of blood tests that covered white blood count (WBC), hemoglobin, platelet count, international normalized ratio, C-reactive protein (CRP), and culture were obtained. We also classify IPA into primary or secondary types based on the presence of other sources of infection. Primary IPA usually originates within the muscle itself without any other infection focus, while secondary IPA occurs as a result of an infection that spreads from a nearby organ or tissue.

The primary outcome was in-hospital mortality. The secondary outcomes were the length of hospital stay (LOS) longer than 30 days and IPA recurrence. Patients were followed up with for one year to identify IPA recurrence, which was determined by reviewing imaging findings and surgical records in the EMRs in both outpatient and inpatient settings.

### 2.3. Statistical Analysis

Patients were divided into survivors and non-survivors. Patient characteristics, comorbidities, initial presentations, laboratory findings, organisms, treatment modalities, and outcomes were reported as numbers (percentages) for the categorical variables and mean ± standard deviation (SD) for the continuous variables. For the categorical variables, the characteristics of the survivors and non-survivor were compared using the Chi-square test or Fisher’s exact test as appropriate. For the continuous variables, independent Student’s *t*-tests were used for the normally distributed variables, and Mann–Whitney U-tests were used for the skewed variables. The optimal cutoff values of IPA diameter for treatment modality were determined using Youden’s index from ROC curve analysis. To identify the predictors for in-hospital mortality, the length of hospital stays (LOS) longer than 30 days, and IPA recurrence, a univariate logistic regression was first performed, and the statistically significant risk factors (*p* < 0.05) were then selected and used in a multivariate logistic regression model. All analyses were performed using SPSS software v26.0 (SPSS Inc., Chicago, IL, USA). A two-sided *p* value of <0.05 was considered statistically significant.

## 3. Results

### 3.1. Patient Characteristics between Survivors and Non-Survivors

A total of 176 patients met the inclusion criteria and were included in the study. The patient characteristics are presented in Table 1. The number of patients who survived hospitalization was 163 (92.6%), and the number who did not survive hospitalization was 13 (7.4%). The distributions of age and sex did not differ between survivors and non-survivors. Twelve (6.8%) patients were bedridden, with significantly more patients in the non-survivor group (23.1 vs. 5.5%, *p =* 0.047). The non-survivor group showed a significantly higher rate of congestive heart failure (30.8% vs. 3.1%, *p* = 0.002) and renal replacement therapy (46.2% vs. 9.2%, *p* = 0.001) than the survivor group. The most frequent presenting symptom was flank or back pain (*n* = 130, 73.9%) and the traditionally described psoas abscess triad of fever, flank or back pain, and limp were present only in 45 (25.6%) patients. Flank or back pain was significantly less frequent in the non-survivor group (38.5 vs. 76.7%, *p* = 0.006). Multiple lobulated IPA was observed in 89 (50.6%) patients, while gas-forming IPA were found in 71 (40.3%) patients. The average maximal IPA diameter was 7.4 ± 4.1 cm. Regarding the laboratory findings, a significantly higher proportion of patients in the non-survivor group had thrombocytopenia (53.8% vs. 17.8%; *p* = 0.006).

### 3.2. Distribution of IPA According to Origin

IPA was of primary origin in 50 patients (28.4%) and of secondary origin in 126 (71.6%). Skeletal origin was the most common source of secondary IPA (*n* = 92, 52.3%), followed by intra-abdominal origin (*n* = 12, 6.8%), urinary tract origin (*n* = 9, 5.1%), cardiovascular origin (*n* = 7, 4%), and soft tissue origin (*n* = 6, 3.4%) (Table 2). Of the skeletal origin, 79 (44.9%) patients had spondylitis and 14 (8%) had septic arthritis. Of the intra-abdominal origin, four (2.3%) patients had intestinal perforation, four (2.3%) had appendicitis, two (1.1%) had an enteric fistula, one (0.6%) had intestinal ischemia, one (0.6%) had diverticulitis, one (0.6%) had colon cancer, and one (0.6%) had endometrial cancer. Of the urinary tract origin, five (2.8%) had pyelonephritis, and four (2.3%) had renal abscess. Of the cardiovascular origin, three (1.7%) had an infected aortic aneurysm (1.7%), three (1.7%) had infective endocarditis, and one (0.6%) had an abdominal aortic aneurysm post stent insertion. Of the soft tissue origin, three (1.7%) had pressure sore, two (1.1%) had necrotizing fasciitis, and one (0.6%) had wound infection.

### 3.3. Microbiology Results

Blood culture was performed in all 176 patients to identify the causative pathogen, and 93 (52.8%) patients showed evidence of bacteremia (Table 3). The most common pathogens were Gram-positive cocci (*n* = 72, 77.4%), including methicillin-susceptible *S. aureus* (MSSA) (*n* = 41, 44.1%), methicillin-resistant *S. aureus* (MRSA) (*n* = 15, 16.1%), Coagulase-negative staphylococcus (CoNS) (*n* = 6, 6.5%), and Group B Streptococcus (*n* = 3, 3.2%), followed by Gram-negative bacilli, including Escherichia coli (*n* = 6, 6.5%), Klebsiella pneumonia (*n* = 6, 6.5%), and Salmonella Group B (*n* = 2, 2.2%). Abscess culture was obtained in 120 patients who underwent PCD or surgery, and the results were positive in 94 (78.3%) of them. Gram-positive cocci were the dominant causative pathogen in 48 (51.1%) patients, including MSSA in 26 (27.7%) patients, MRSA in 14 (14.9%) patients, Coagulase-negative staphylococcus (CoNS) in 3 (3.2%) patients, and Group B Streptococcus in 2 (2.1%) patients, followed by Gram-negative bacilli in 23 (24.5%), including Escherichia coli in 10 (10.6%) patients, Klebsiella pneumonia in 7 (7.4%), Salmonella Group B in 2 (2.1%) patients, and polymicrobial flora in 22 (23.4%) patients.

### 3.4. Treatment and Outcomes

**Figure 2:**
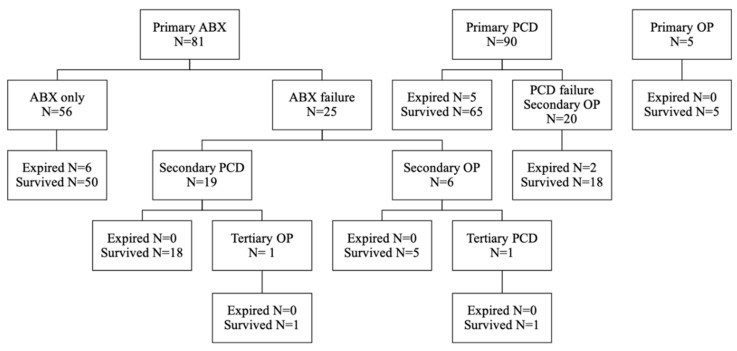
Treatment outcomes with primary antibiotics, percutaneous drainage, and operation in patients with iliopsoas abscess. ABX: antibiotics; PCD: percutaneous drainage; OP: operation.

Each patient received intravenous antibiotics once IPA was diagnosed. Eighty-eight (50%) patients underwent PCD, 32 (18.2%) patients underwent surgical debridement, and 56 (31.8%) patients received antibiotics alone. Overall, 81 (46.0%) patients received primary antibiotic treatment. Of these patients, 50 survived hospitalizations without an interventional procedure, six passed away, and 25 failed the initial antibiotic treatment with additional intervention. The failure rate of antibiotics-only treatment was 38.3% (31/81). Ninety (51.1%) patients underwent primary PCD, of whom 65 survived, five passed away, and 20 failed PCD with additional salvage surgery. The failure rate of primary PCD was 27.8% (25/90). In contrast, five patients underwent primary surgical debridement, and all of them survived with a failure rate of 0% (0/6) (Figure 2). When the abscess diameter was larger than 8cm, primary PCD or surgery was significantly more frequently performed than primary antibiotics in patients with IPA (41.7% vs. 19.6%, *p* = 0.004). Among all the patients with IPA, 86 (48.9%) and 30 (17%) patients presented with sepsis and septic shock, with the percentage being significantly higher in the non-survivors than in the survivors (100% vs. 44.8%, *p* < 0.001; 92.3 vs. 11%, *p* < 0.001). The mean LOS was 32.4 ± 26.9 days. There were 38 (21.6%) patients admitted to the intensive care units, and 13 (7.4%) passed away during hospitalization (Table 4). During a median follow-up period of 9.4 months (IQR: 3.0–28.3), 34 out of 176 patients (19.2%) experienced recurrent IPAs. Among them, the median time to recurrence was 76 days (IQR: 51–196) after the diagnosis of index IPA.

### 3.5. Univariate and Multivariate Analyses of Predictors of In-Hospital Mortality, Longer LOS, and Recurrence

To identify the predictors of in-hospital mortality, we employed univariate Cox regression followed by multivariate Cox regression to create two separate models. Model A consisted of all relevant variables but did not include septic shock. In contrast, Model B included septic shock and the same variables as Model A, except for systolic blood pressure, respiratory rate (RR), and platelet count, which are components used to diagnose sepsis [13]. In multivariate Cox Model A, age > 65 (year) (HR = 5.12; CI 1.03–25.53; *p* = 0.046), congestive heart failure (HR = 5.13; CI 1.29–20.45; *p* = 0.021), and platelet < 150 (10^3^/μL) (HR = 9.26; CI 2.59–33.09; *p* = 0.001) were statistically significant predictors of in-hospital mortality. Multivariate predictors in Model B included age > 65 (year) (HR = 5.12; CI 1.03–25.53; *p* = 0.046) and septic shock (HR = 61.90; CI 7.37–519.46; *p* < 0.001) (Table 5). To determine the predictors of longer LOS, patients were stratified into two groups—LOS longer than 30 days (*n* = 81, 46%) and LOS shorter than 30 days (*n* = 95, 54%). The multivariate analysis indicated that RR > 22 (breaths/min) (OR = 3.33; CI 1.09–10.20; *p* = 0.035), gas-forming abscess (OR = 2.15; CI 1.03–4.46; *p* = 0.041), chronic kidney disease (OR = 5.23; CI 1.91–14.31; *p* = 0.001), skeletal origin (OR = 4.92; CI 1.98–12.22; *p* = 0.001), PCD (OR = 2.95; CI 1.01–8.26; *p* = 0.048), and surgery (OR = 3.77; CI 1.58–8.99; *p* = 0.003) were all statistically significant independent predictors of LOS longer than 30 days (Table 6). For recurrence, the univariate Cox regression identified a single statistically significant predictor for HIV infection (HR = 4.69; CI 1.41–15.57; *p* = 0.012), and the multivariate regression analysis was thereby not performed (Table 7).

## 4. Discussion

This retrospective study examined the clinical characteristics in patients with iliopsoas abscess and analyzed the predictors of in-hospital outcomes—in-hospital mortality, LOS—and recurrence, which have not been previously reported. Our results showed that patients with an advanced age, congestive heart failure, and thrombocytopenia had a higher risk of in-hospital mortality. The independent predictors of LOS longer than 30 days were tachypnea, gas-forming abscess, chronic kidney disease, skeletal origin, and the need of PCD or surgery, and the independent predictor of IPA recurrence was HIV infection.

Similar to the rate of 30% in the study by Chern et al. [4], the traditionally described psoas abscess triad—fever, flank/back pain, and limp [2]—presented in around only one-fourth of the patients in our study (45 patients, 25.6%), and flank or back pain was especially less presented in the non-survivor group (38.5 vs. 76.7%, *p* = 0.006). This is compatible with the results demonstrated by Hamano et al., which showed that the classic presentation was significantly infrequent in patients with septic shock than those without (*p* = 0.012) [13]. This observation signifies that the timely recognition of IPA was difficult in the patients without the typical presentation, which can lead to poor prognosis.

Because of the non-specific symptoms and signs of IPA, it is often difficult to diagnose, and this can result in life-threatening situations [3]. Various studies have analyzed the clinical features and risk factors associated with IPA [3,14,15,16]. However, owing to the rarity of the disease, the majority of research related to IPA presented a limited number of patients and only in the form of case reports and short case series [4,10,17]. To the best of our knowledge, our multicenter-based study had the largest patient number in the current literature. Moreover, this is the first study to report the predictors for poor outcomes, which consist of not only in-hospital mortality, but also length of stay (LOS) and recurrence, in patients with IPA. With a longer LOS, patients with IPA may undergo a longer period of antibiotic therapy and management of potential complications. IPA recurrence may cause further antibiotics resistance or dissemination of the infection to adjacent organs. Patients with recurrent IPA may go through more aggressive treatment modalities and a longer period of treatment. The aforementioned consequence implies a greater healthcare burden for the hospital and higher medical expenses for the patients. To ensure effective and efficient care, it is crucial to take these factors into account when assessing each patient’s risk and determining the optimal treatment plan. By recognizing these associated consequences, we can improve patient outcomes while reducing healthcare costs.

IPA is classified into primary and secondary origins by whether there is an initial site other than the iliopsoas compartment. Some literature viewed skeletal origin as the dominant infectious source. In 2009, Lopez et al. collected 124 cases, with 78.2% having secondary IPA, and most abscesses are secondary to a skeletal source [2]. The studies inspected by Hsieh et al. in Taiwan, Wong et al. in Hong Kong, and Kim et al. in Korea demonstrated a similar pattern, with the dominance of secondary IPA and the most common infection origins being skeletal [3,14,16,18,19]. Our results reflected an identical trend with the majority of IPA cases being of secondary origin (71.6%), and skeletal origin being the most common source of secondary IPA (*n* = 92, 52.3%), followed by intra-abdominal origin (*n* = 12, 6.8%). A study in Argentina illustrated that skeleton was the leading origin of IPA [14] and suggested that this could be explained by the low incidence of inflammatory bowel disease in their population, which is compatible with the pattern in our study.

The microbiological corroboration rate in IPA patients was 52.8% (93/176) with positive blood culture and 78.3% (94/120) with positive pus culture in our study. The causative organisms were predominantly Gram-positive bacteria, with *S. aureus* attributable to more than 60% of these cases, and mostly isolated from primary and skeletal origin. Of *S. aureus* cases, MRSA was accountable in 26.8% (15/56) of blood cultures, and 35% (14/40) of abscess aspirate cultures; both of these prevalence rates were comparable to that of the general population [3,16,20]. Whereas some of the literature suggested that empiric coverage of MRSA is not warranted for all IPA patients due to the low frequency [2,14], other recent articles indicated a significant increase in MRSA isolated from IPA [16,20]. This may be due to several reasons, such as drug-resistant organisms, repeat surgery, and increasing immunocompromised hosts [7,20,21]. Even though most cases of primary and skeletal IPA were caused by Gram-positive cocci, IPA from urinary or gastrointestinal origins usually involved the isolation of Gram-negative bacilli, and the abscess aspirate culture revealed that more than half of these cases involved polymicrobial infections [22]. Therefore, the timely initiation of empirical antimicrobial therapy with broad-spectrum coverage for iliopsoas abscess is crucial, and regional epidemiologic patterns of susceptibility should always be considered.

Antibiotic treatment combined with abscess drainage is the treatment of choice for IPA. In our cohort, 45.5% patients received primary antibiotic treatment in our study, of whom only 21.8% (50/80) survived hospitalization without an interventional procedure. The failure rate of antibiotics-only treatment was 37.5% (30/80) with six patients who have passed away. Some authors have advocated that targeted antibiotics may be adequate to treat abscesses less than 6 cm [23]. Our study showed that PCD or surgery was significantly more frequently performed than antibiotics alone in patients with IPA when the abscess was larger than 8 cm. Accordingly, we recommend careful evaluation for timely abscess drainage—including surgical intervention or PCD—in the presence of co-existent bowel lesions or retroperitoneal abnormalities, phlegmonous involvement of muscle without liquefaction, multiloculated, gas-forming, or large abscess [2,3,7,9,24,25].

Few studies have analyzed the risk factors for mortality, but some previous research revealed that advanced age, thrombocytopenia, elevated serum creatinine level, bacteremia, and cardiovascular disease were associated with in-hospital mortality [2,3,16,26,27,28]. Our study indicated that advanced age, congestive heart failure, thrombocytopenia, and septic shock were significant independent risk factors for in-hospital mortality. Thrombocytopenia is not only an indicator of severe sepsis, but also suspends the administration of drainage [27]. The severity of the existing physical condition and clinical status (e.g., advanced age, congestive heart failure) may affect the prognosis in patients with IPA. The development of septic shock significantly increases the risk of mortality, which is attributed to several factors, including multiple organ dysfunction, immune system response, and treatment challenges [13,29]. For patients at a higher risk of mortality, a more aggressive therapeutic approach and more frequent monitoring should be considered in addition to empiric antibiotic treatment.

Our study revealed that tachypnea, gas-forming abscess, chronic kidney disease, skeletal origin, and the need of PCD or surgery are associated with LOS longer than 30 days. No studies have assessed the risk factors for longer LOS in patients with IPA. Although the relation between tachypnea and prognosis has not been reported, some studies have reported that at triage certain vital signs, such as low blood pressure, were associated with poor prognosis [27]. Tachypnea may be related to acidosis or respiratory failure and revealed the severity of sepsis, which would complicate the hospital course [27]. One study conducted by Hsieh et al. revealed that gas-forming IPA frequently requires aggressive drainage and, consequently, contributes to longer LOS [3]. Several studies have demonstrated that an elevated serum creatinine level or dialysis status was associated with poor prognosis [2,3,16,26,27,29,30]. Chronic kidney disease results in impairment of the immune system and aggravates the disease [31,32,33]. For patients with IPA from secondary origin, the infectious origin needs to be treated in addition to IPA. The standard treatment duration for skeletal infections, such as osteomyelitis or spondylitis, is typically longer than that of other types of infections, often lasting six to eight weeks. This is likely the reason behind the correlation between infections of skeletal origin and a longer LOS.

Our study was the first to examine the predictors of IPA recurrence. The results of our analysis indicated that HIV infection was the predictor for IPA recurrence. The study by Lopez et al. highlighted the specific clinical features of IPA in patients with HIV infection [21]. The immunosuppression in this population leads to a high susceptibility to opportunistic infections. The difficulty of completely eliminating pathogens, combined with their weakened immune status, means that patients with HIV infection are at a heightened risk for IPA recurrence.

## 5. Limitations

This study has several limitations. First, its retrospective design resulted in missing data and an uneven collection of clinical variables, such as a lack of abscess aspirate culture for all patients. Second, the rarity of IPA resulted in a small sample size, which might have limited the statistical power and generalizability of our findings. Additionally, the limited number of mortality events could disproportionately influence the model’s estimates of the impact of specific variables. Finally, our study was conducted in Taiwan, a monoethnic country where most cases were Asian, so our results may not be representative of the broader population. Further prospective multi-center studies with large sample sizes would be needed to further verify our findings.

## 6. Conclusions

Iliopsoas abscess is a medical emergency requiring early recognition for the initiation of timely treatment and the reduction of the associated morbidity and mortality. Patients suffering from iliopsoas abscess with advanced age, congestive heart failure, thrombocytopenia, and septic shock had a significantly higher risk of mortality. Tachypnea, gas-forming abscess, chronic kidney disease, IPA from the skeletal origin, and the need of PCD or surgery were associated with a longer LOS. Patients with HIV infection were vulnerable to the recurrence of IPA. The recognition of these associated factors allows for risk stratification and the determination of the optimal treatment plan in patients with iliopsoas abscess.

## Figures and Tables

**Table 1:** Demographics, clinical characteristics, laboratory results, and treatment modalities by survival status in patients with iliopsoas abscess.

Variable	Total (*n* = 176)	Survivor (*n* = 163)	Non-Survivor (*n* = 13)	*p* Value
Age (year)	63.0 ± 16.4	61.9 ± 13.6	76.2 ± 13.3	0.002 *
Male	114 (64.8)	107 (65.6)	7 (53.8)	0.385
Systolic blood pressure (mmHg)	130.3 ± 31.1	129.8 ± 30.5	136.9 ± 38.8	0.425
Diastolic blood pressure (mmHg)	73.1 ± 17.1	72.9 ± 16.9	76.0 ± 20.2	0.527
Heart rate (beats/min)	101.7 ± 19.5	101.8 ± 19.3	100.5 ± 23.0	0.823
Body temperature (°C)	37.1 ± 1.0	37.1 ± 1.1	36.5 ± 0.9	0.029 *
Respiratory rate (breaths/min)	19.4 ± 2.9	19.2 ± 2.8	21.2 ± 3.5	0.020 *
Bedridden status	12 (6.8)	9 (5.5)	3 (23.1)	0.047 *
Mechanical ventilation	32 (18.2)	22 (13.5)	10 (76.9)	<0.001 *
Intensive care unit admission	38 (21.6)	28 (17.2)	10 (76.9)	<0.001 *
Renal replacement therapy	21 (11.9)	15 (9.2)	6 (46.2)	0.001 *
Length of hospital stay (day)	32.4 ± 26.9	33.0 ± 26.6	26.1 ± 30.3	0.371
Recurrence	34 (19.3)	34 (20.9)	0 (0)	
Sepsis	86 (48.9)	73 (44.8)	13 (100)	<0.001 *
Septic shock	30 (17.0)	18 (11)	12 (92.3)	<0.001 *
**Abscess features**				
Gas-forming	71 (40.3)	63 (38.7)	8 (61.5)	0.105
Multiple lobulated	89 (50.6)	83 (50.9)	6 (46.2)	0.781
Size (cm)	7.4 ± 4.1	7.4 ± 4.0	8.1 ± 3.3	0.554
Involvement				0.996
Right	67 (38.1)	62 (38.0)	5 (38.5)	
Left	56 (31.8)	52 (31.9)	4 (30.8)	
Bilateral	53 (30.1)	49 (30.1)	4 (30.8)	
**Initial presentations**				
Fever	96 (54.5)	92 (56.4)	4 (30.8)	0.074
Abdominal pain	40 (22.7)	35 (21.5)	5 (38.5)	0.175
Flank/back pain	130 (73.9)	125 (76.7)	5 (38.5)	0.006 *
Limp	90 (51.1)	86 (52.8)	4 (30.8)	0.127
Mass	3 (1.7)	3 (1.8)	0 (0)	1.000
Malaise	70 (39.8)	62 (38.0)	8 (61.5)	0.096
**Comorbidities**				
Hypertension	83 (47.2)	78 (47.9)	5 (38.5)	0.514
Diabetes mellitus	66 (37.5)	62 (38.0)	4 (30.8)	0.769
CAD	15 (8.5)	14 (8.6)	1 (7.7)	1.000
CKD	32 (18.2)	28 (17.2)	4 (30.8)	0.258
CHF	9 (5.1)	5 (3.1)	4 (30.8)	0.002 *
Malignancy	21 (11.9)	19 (11.7)	2 (15.4)	0.656
Prior stroke	11 (6.3)	10 (6.1)	1 (7.7)	0.581
Intravenous drug abuse	21 (11.9)	21 (12.9)	0 (0)	0.370
HIV infection	4 (2.3)	4 (2.5)	0 (0)	1.000
Liver cirrhosis	12 (6.8)	11 (6.7)	1 (7.7)	1.000
Prior TB history	1 (0.6)	3 (1.8)	0 (0)	1.000
**Imaging modalities**				
Computed tomography	172 (97.7)	159 (97.5)	13 (100.0)	1.000
Magnetic resonance imaging	71 (40.3)	65 (39.9)	6 (46.2)	0.657
**Blood test**				
White blood count (10^3^/μL) *n* = 17	16.6 ± 9.6	16.9 ± 9.8	13.1 ± 5.9	0.178
Hemoglobin (g/dL) *n* = 175	11.3 ± 5.6	11.4 ± 5.7	10.8 ± 3.4	0.552
Platelet (10^3^/μL) *n* = 46	264.8 ± 144.0	273.1 ± 142.1	162.5 ± 133.0	0.007 *
INR *n* = 18	1.2 ± 0.4	1.2 ± 0.4	1.3 ± 0.3	0.354
Creatinine (mg/dL) *n* = 176	2.1 ± 5.3	2.1 ± 5.5	2.7 ± 2.2	0.730
C-reactive protein (mg/L) *n* = 41	187.8 ± 121.9	189.4 ± 120.0	166.6 ± 151.9	0.534
Positive culture *n* = 176	93 (52.8)	85 (52.1)	8 (61.5)	0.514
**Treatment**				
Antibiotics alone	56 (31.8)	50 (30.7)	6 (46.2)	0.352
Percutaneous drainage	88 (50.0)	83 (50.9)	5 (38.5)	0.387
Surgery	32 (18.2)	30 (18.4)	2 (15.4)	1.000

Count data are expressed as number (percentage) and continuous values are expressed as mean ± SD. CAD: coronary artery disease; CKD: chronic kidney diseases; CHF: congestive heart failure; HIV: human immunodeficiency virus; TB: tuberculosis; INR: international normalized ratio. * *p* value < 0.05.

**Table 2:** Types and origins of iliopsoas abscess by survival status.

Variable	Total (*n* = 176)	Survivor (*n* = 163)	Non-Survivor (*n* = 13)	*p* Value
Primary IPA	50 (28.4)	45 (27.6)	5 (38.5)	0.523
Secondary IPA				
Skeletal origin	92 (52.3)	88 (54.0)	4 (30.8)	0.107
Spondylitis	79 (44.9)	75 (46.0)	4 (30.8)	
Septic arthritis	14 (8.0)	14 (8.6)	0 (0)	
Intra-abdominal origin	12 (6.8)	11 (6.7)	1 (7.7)	1.000
Intestinal perforation	4 (2.3)	3 (1.8)	1 (7.7)	
Intestinal ischemia	1 (0.6)	1 (0.6)	0 (0)	
Enteric fistula	2 (1.1)	2 (1.2)	0 (0)	
Diverticulitis	1 (0.6)	1 (0.6)	0 (0)	
Appendicitis	4 (2.3)	4 (2.5)	0 (0)	
Colon cancer	1 (0.6)	1 (0.6)	0 (0)	
Endometrial cancer	1 (0.6)	1 (0.6)	0 (0)	
Urinary tract origin	9 (5.1)	7 (4.3)	2 (15.4)	0.135
Pyelonephritis	5 (2.8)	3 (1.9)	2 (15.4)	
Renal abscess	4 (2.3)	4 (2.5)	0 (0)	
Soft tissue origin	6 (3.4)	5 (3.1)	1 (7.7)	0.373
Necrotizing fasciitis	2 (1.1)	2 (1.2)	0 (0)	
Wound infection	1 (0.6)	1 (0.6)	0 (0)	
Pressure sore	3 (1.7)	2 (1.2)	1 (7.7)	
Cardiovascular origin	7 (4.0)	7 (4.3)	0 (0)	1.000
Abdominal aortic aneurysm post stent insertion	1 (0.6)	1 (0.6)	0 (0)	
Infected aortic aneurysm	3 (1.7)	3 (1.8)	0 (0)	
Infective endocarditis	3 (1.7)	3 (1.8)	0 (0)	

Count data are expressed as number (percentage) and continuous values are expressed as mean ± SD. IPA: iliopsoas abscess.

**Table 3:** Microbiology results of blood cultures and tissue cultures stratified by primary and secondary origin.

	Total		Primary		Secondary									
					Skeletal		GI		GU		Soft Tissue		CV	
	*n*	(%)	*n*	(%)	*n*	(%)	*n*	(%)	*n*	(%)	*n*	(%)	*n*	(%)
**Blood culture**	93		25		56		1		4		4		3	
**Gram-positive**														
MSSA	41	(44.1)	13	(52.0)	26	(46.4)					1	(25.0)	1	(33.3)
MRSA	15	(16.1)	3	(12.0)	9	(16.1)			1	(25.0)	1	(25.0)	1	(33.3)
CoNS	6	(6.5)	1	(4.0)	2	(3.6)			1	(25.0)	2	(50.0)		
GBS	3	(3.2)	1	(4.0)	2	(3.6)								
Streptococcus intermedius	2	(2.2)			2	(3.6)								
Group D Streptococcus	1	(1.1)	1	(4.0)										
Streptococcus gordonii	1	(1.1)			1	(1.8)								
Streptococcus mitis	1	(1.1)	1	(4.0)										
Anaerococcus sp.	1	(1.1)			1	(1.8)								
Peptostreptococcus sp.	1	(1.1)	1	(4.0)										
**Gram-negative**														
Klebsiella pneumoniae	6	(6.5)	2	(8.0)	4	(7.1)								
Escherichia coli	6	(6.5)			5	(8.9)			1	(25.0)				
Salmonella enterica serogroup B	2	(2.2)			1	(1.8)							1	(33.3)
Salmonella enterica serogroup D	1	(1.1)					1	(100.0)						
Serratia marcescens	1	(1.1)							1	(25.0)				
Actinobacillus actinomycetemcomitans	1	(1.1)	1	(4.0)										
Aeromonas spp.	1	(1.1)			1	(1.8)								
Burkholderia pseudomallei	1	(1.1)			1	(1.8)								
**Fungus**														
Candida albicans	1	(1.1)	1	(4.0)										
**Polymicrobial**	1	(1.1)			1	(1.8)								
**Tissue culture**	94		25		50		7		6		4		2	
**Gram-positive**														
MSSA	26	(27.7)	7	(28.0)	19	(38.0)								
MRSA	14	(14.9)	6	(24.0)	7	(14.0)			1	(16.7)				
CoNS	3	(3.2)			3	(6.0)								
GBS	2	(2.1)	1	(4.0)	1	(2.0)								
Enterococcus faecalis	1	(1.1)			1	(2.0)								
Streptococcus intermedius	1	(1.1)			1	(2.0)								
Viridans streptococcus	1	(1.1)	1	(4.0)										
**Gram-negative**														
Escherichia coli	10	(10.6)			5	(10.0)	2	(28.6)	2	(33.3)	1	(25.0)		
Klebsiella pneumoniae	7	(7.4)	4	(16.0)	3	(6.0)								
Salmonella enterica serogroup B	2	(2.1)			2	(4.0)								
Salmonella enterica serogroup D	1	(1.1)			1	(2.0)								
Bacteroides fragilis	1	(1.1)			1	(2.0)								
Pseudomonas aeruginosa CR strain	1	(1.1)			1	(2.0)								
Serratia marcescens	1	(1.1)			1	(2.0)								
**Polymicrobial**	22	(23.4)	6	(24.0)	3	(6.0)	5	(71.4)	3	(50.0)	3	(75.0)	2	(100.0)
**Tuberculosis**	1	(1.1)			1	(2.0)								

MSSA: methicillin-susceptible Staphylococcus aureus; MRSA: methicillin-resistant Staphylococcus aureus; CoNS: Coagulase-negative staphylococcus; GBS: Group B Streptococcus; GI: gastrointestinal; GU: genitourinary; CV: cardiovascular.

**Table 4:** Description of patients with iliopsoas abscess who passed away during hospitalization.

No.	Age	Sex	Underlying Diseases	Clinical Presentation	Blood Culture	Pus Culture	Location	Percutaneous Drainage	Surgical Debridement	Antibiotics Treatment
1	78	M	DM, HTN, ESRD, gout, HIVD	abdominal pain	N/A	Mycobacterium tuberculosis complex	L	Y	Y	Teicoplanin + Ceftriaxone (10), Piperacillin/Tazobactam + Daptomycin (8), Teicoplanin + Piperacillin/Tazobactam (7), Rifinah + Pyrazinamide (26), Teicoplanin + Cefepime (5)
2	50	F	cirrhosis	fever, abdominal pain, seizure	N/A	N/A	R	N	N	Piperacillin/Tazobactam (5)
3	87	F	HTN, asthma	flank/back pain, limp, cold sweating, chillness, SOB, conscious unclear, urine/stool retention	MSSA	N/A	B	N	N	Teicoplanin + Cefoperazone/Sulbactam + Clindamycin (2), Cefepime + Vancomycin + Metronidazole (3)
4	81	F	dementia, bedridden, s/p pacemaker	fever, malaise	MSSA	MSSA	L	Y	N	Piperacillin/Tazobactam (1), Cefazolin + Ciprofloxacin (6), Vancomycin + Piperacillin/Tazobactam (4), Oxacillin (9)
5	87	F	CHF, CKD	fever, conscious drowsy	N/A	N/A	R	Y	N	Piperacillin/Tazobactam (21), Levofloxacin (3), Cefepime (7), Tigecycline
6	74	M	DM	fever, abdominal pain, malaise, anorexia, body weight loss, disoriented	N/A	Klebsiella pneumoniae	L	N	N	Ceftriaxone + Metronidazole (6), Teicoplanin + Imipenem/Cilastatin (1)
7	82	M	HTN, CVA, bedridden, CO intoxication-related HIE, SDH/SAH/EDH Hx	malaise, limbs edema	MSSA	MSSA, Proteus mirabilis, Escherichia coli	B	N	N	Piperacillin/Tazobactam (6), Teicoplanin (3)
8	66	M	DM, HTN, hyperparathyroidism	flank/back pain, conscious disturbance, SOB	Escherichia coli	N/A	R	N	N	Piperacillin/Tazobactam + Vancomycin(1), Ertapenem (1), Doripenem + Doxycycline (9)
9	83	F	CHF, DM, ESRD, breast cancer, Af	abdominal pain, left calf erythema, conscious drowsy	MSSA	MSSA	L	Y	N	Imipenem/Cilastatin + Vancomycin (5), Oxacillin (8), Ceftriaxone(5), Ertapenem (2)
10	83	M	CHF, HTN, CKD, gout	flank/back pain, limp	MRSA	N/A	B	N	N	Ertapenem(2), Daptomycin(2), Teicoplanin + Cefepime(10), Teicoplanin (4), Teicoplanin + Cefepime (3), Teicoplanin + Levofloxacin (8), Vancomycin (23), Vancomycin + Meropenem (3), Ertapenem (5), Daptomycin + Meropenem (15), Levofloxacin + Micafungin + Daptomycin (8), Piperacillin/Tazobactam+ Amikacin + Amphotericin B (4)
11	89	F	CHF, VHD, Af	flank/back pain, limp	N/A	Escherichia coli, Bacteroides fragilis, Streptococcus anginosus, Bacteroides thetaiotaomicron, Yeast-like	R	Y	Y	Piperacillin/Tazobactam (11), Ampicillin + Sulbactam (10), Piperacillin/Tazobactam (3)
12	49	M	oropharyngeal cancer	flank/back pain, limp, malaise	MSSA	Escherichia coli, Enterococcus faecium, Bacteroides fragilis, Bacteroides thetaiotaomicron	B	Y	Y	Cefoperazone/Sulbactam + Vancomycin (2), Meropenem + Teicoplanin (2), Doxycycline + Ceftriaxone + Vancomycin (3), Oxacillin (12), Flomoxef (3), Ceftriaxone (6), Ertapenem (11), Ciprofloxacin (6), Teicoplanin (4), Ceftazidime (5), Teicoplanin + Ampicillin + Sulbactam + Doripenem (4) Oxacillin + Doripenem (5), Oxacillin + Metronidazole (10), Oxacillin + Piperacillin/Tazobactam (14)
13	81	M	asthma	abdominal pain	Peptostreptococcus sp	Bacteroides sp, Pseudomonas aeruginosa	R	Y	N	Ceftriaxone, Metronidazole (1)

N/A: not available; L: left; R: right; B: both; Y: yes; N: no; DM: diabetes mellitus; HTN: hypertension; ESRD: end-stage renal diseases; HIVD: herniated intervertebral disc; CHF: congestive heart failure; CKD: chronic kidney diseases; CAD: coronary artery diseases; CVA: cerebrovascular accident; HIE: hypoxic ischemic encephalopathy; SDH: subdural hemorrhage; SAH: subarachnoid hemorrhage; EDH: epidural hemorrhage; Af: atrial fibrillation; VHD: valvular heart diseases; MSSA: methicillin-susceptible Staphylococcus aureus; MRSA: methicillin-resistant Staphylococcus aureus.

**Table 5:** Univariate and multivariate analyses of predictors of in-hospital mortality with Cox proportional hazards model.

	Univariate		Multivariate (Model A)		Multivariate (Model B)	
	HR (95%CI)	*p* Value	HR (95%CI)	*p* Value	HR (95%CI)	*p* Value
Age > 65 (year)	6.68 (1.48, 30.16)	0.013	5.12 (1.03,25.53)	0.046 *	6.06 (1.22,30.23)	0.028 *
Male	0.62 (0.21, 1.84)	0.389				
BT > 38 or <36 °C	0.48 (0.11, 2.18)	0.343				
Heart rate > 100 (beats/min)	1.08 (0.36, 3.22)	0.887				
SBP < 100 (mmHg)	1.18 (0.26, 5.29)	0.836				
RR > 22 (breaths/min)	4.98 (1.63, 15.23)	0.005	1.41 (0.34, 5.95)	0.637		
Septic shock	71.49 (9.28, 550.63)	<0.001			61.90 (7.37, 519.46)	<0.001 *
Renal replacement therapy	6.87 (2.31, 20.44)	0.001	3.81 (0.94, 15.49)	0.062	1.21 (0.39, 3.73)	0.745
Bedridden status	4.42 (1.22, 16.07)	0.024	2.37 (0.56, 10.08)	0.243	1.37 (0.33, 5.70)	0.669
Gas-forming abscess	2.44 (0.80, 7.44)	0.119				
Hypertension	0.69 (0.23, 2.11)	0.517				
Diabetes mellitus	0.73 (0.23, 2.38)	0.604				
Congestive heart failure	9.20 (2.82, 29.97)	<0.001	5.13 (1.29, 20.45)	0.021 *	2.04 (0.51, 8.10)	0.313
Coronary artery disease	0.87 (0.11, 6.70)	0.895				
Chronic kidney disease	1.99 (0.61, 6.46)	0.253				
HIV	0.31 (0.04, 2.24)	0.244				
WBC > 11 (10^3^/μL)	0.52 (0.17, 1.54)	0.234				
Hb < 8 (g/dL)	2.33 (0.64, 8.48)	0.198				
Plt < 150 (10^3^/μL)	4.94 (1.66, 14.71)	0.004	9.26 (2.59, 33.09)	0.001 *		
IPA origin						
Primary origin	Reference					
Skeletal origin	0.42 (0.11, 1.55)	0.190				
Intra-abdominal origin	0.81 (0.09, 6.92)	0.846				
Urinary tract origin	2.42 (0.47, 12.46)	0.292				
Soft tissue origin	1.76 (0.21, 15.05)	0.607				
Cardiovascular origin	0.55 (0.03, 11.04)	0.985				
Treatment						
Antibiotics alone	Reference					
Percutaneous drainage	0.55 (0.11, 2.74)	0.467				
Surgery	0.51 (0.16, 1.67)	0.266				

HR: hazard ratio; 95% CI: 95% confidence interval; BT: blood temperature; SBP: systolic blood pressure; RR: respiratory rate; HIV: human immunodeficiency virus; WBC: white blood cell; Hb: hemoglobin; Plt: platelet. * *p* value < 0.05.

**Table 6:** Univariate and multivariate analyses of predictors of length of hospital stay longer than 30 days with logistic regression model.

	Univariate		Multivariate	
	OR (95%CI)	*p* Value	OR (95%CI)	*p* Value
Age > 65 (year)	1.63 (0.89, 2.96)	0.112		
Male	1.06 (0.57, 1.96)	0.866		
BT > 38 or <36 °C	0.99 (0.51, 1.93)	0.975		
Heart rate > 100 (beats/min)	1.21 (0.67, 2.20)	0.521		
SBP < 100 (mmHg)	0.82 (0.34, 1.95)	0.645		
RR > 22 (breaths/min)	2.86 (1.10, 7.40)	0.031	3.33 (1.09, 10.20)	0.035 *
Renal replacement therapy	2.08 (0.82, 5.30)	0.125		
Bedridden status	0.83 (0.25, 2.71)	0.754		
Gas-forming abscess	2.45 (1.32, 4.54)	0.004	2.15 (1.03, 4.46)	0.041 *
Hypertension	0.90 (0.49, 1.62)	0.716		
Diabetes mellitus	1.42 (0.77, 2.63)	0.258		
Congestive heart failure	1.50 (0.39, 5.77)	0.558		
Coronary artery disease	1.85 (0.63, 5.45)	0.262		
Chronic kidney disease	3.79 (1.64, 8.77)	0.002	5.23 (1.91, 14.31)	0.001 *
HIV	1.18 (0.16, 8.55)	0.872		
WBC > 11 (10^3^/μL)	1.50 (0.78, 2.85)	0.222		
Hb < 8 (g/dL)	1.34 (0.54, 3.33)	0.534		
Plt < 150 (10^3^/μL)	1.87 (0.89, 3.92)	0.099		
IPA origin				
Primary origin	Reference		Reference	
Skeletal origin	3.82 (1.82, 8.05)	<0.001	4.92 (1.98, 12.22)	0.001 *
Intra-abdominal origin	0.23 (0.03, 1.98)	0.183	0.24 (0.03, 2.18)	0.202
Urinary tract origin	2.06 (0.48, 8.79)	0.330	2.05 (0.38, 11.01)	0.402
Soft tissue origin	1.29 (0.21, 7.83)	0.785	2.52 (0.37, 17.08)	0.343
Cardiovascular origin	6.43 (1.12, 37.07)	0.037	7.18 (0.90, 57.51)	0.063
Treatment				
Antibiotics alone	Reference		Reference	
Percutaneous drainage	2.95 (1.20, 7.26)	0.019	2.95 (1.01, 8.62)	0.048 *
Surgery	2.51 (1.24, 5.09)	0.011	3.77 (1.58, 8.99)	0.003 *

HR: hazard ratio; 95% CI: 95% confidence interval; BT: blood temperature; SBP: systolic blood pressure; RR: respiratory rate; HIV: human immunodeficiency virus; WBC: white blood cell; Hb: hemoglobin; Plt: platelet. * *p* value < 0.05.

**Table 7:** Univariate analysis of predictors of recurrence with Cox proportional hazards model.

	Univariate	
	HR (95%CI)	*p* Value
Age > 65 (year)	1.68 (0.34, 1.37)	0.279
Male	0.89 (0.44, 1.79)	0.733
BT > 38 or <36 °C	0.56 (0.23, 1.36)	0.200
Heart rate > 100 (beats/min)	1.07 (0.55, 2.11)	0.840
SBP < 100 (mmHg)	1.48 (0.61, 3.58)	0.384
RR > 22 (breaths/min)	1.07 (0.38, 3.04)	0.903
Renal replacement therapy	1.03 (0.36, 2.92)	0.958
Bedridden status	2.40 (0.73, 7.88)	0.148
Gas-forming abscess	1.15 (0.58, 2.26)	0.690
Hypertension	1.10 (0.56, 2.15)	0.786
Diabetes mellitus	1.14 (0.58, 2.25)	0.698
Congestive heart failure	0.69 (0.09, 5.05)	0.715
Coronary artery disease	2.14 (0.88, 5.16)	0.092
Chronic kidney disease	0.56 (196, 1.58)	0.271
HIV	4.69 (1.41, 15.57)	0.012 *
WBC > 11 (10^3^/μL)	0.84 (0.41, 1.72)	0.626
Hb < 8 (g/dL)	0.61 (0.15, 2.56)	0.501
Plt < 150 (10^3^/μL)	1.71 (0.80, 3.67)	0.168
IPA origin		
Primary origin	Reference	
Skeletal origin	0.73 (0.32, 1.70)	0.466
Intra-abdominal origin	2.21 (0.72, 6.75)	0.166
Urinary tract origin	1.12 (0.24, 5.31)	0.884
Soft tissue origin	0.03 (0.002, 0.53)	0.978
Cardiovascular origin	1.44 (0.30, 6.80)	0.647
Treatment		
Antibiotics alone	Reference	
Percutaneous drainage	0.66 (0.23, 1.94)	0.454
Surgery	1.02 (0.48, 2.20)	0.952

OR: odds ratio; 95% CI: 95% confidence interval; BT: blood temperature; SBP: systolic blood pressure; RR: respiratory rate; HIV: human immunodeficiency virus; WBC: white blood cell; Hb: hemoglobin; Plt: platelet. * *p* value < 0.05.

## Data Availability

Not applicable.
